# Unveiling a Novel Mechanism in Noise-Induced Hearing Loss: Oxeiptosis-Mediated Regulated Cell Death of Cochlear Hair Cell

**DOI:** 10.1007/s12264-025-01585-z

**Published:** 2026-02-16

**Authors:** Xinyu Zhang, Meihao Qi, Peng Zhang, Zejun Gao, Ziqi Wu, Wenyue Wang, Runqin Yang, Xiaogang An, Fei Lu, Renfeng Wang, Qingwen Zhu, Dingjun Zha

**Affiliations:** 1 Department of Otolaryngology-Head and Neck Surgery, Xijing Hospital, Air Force Medical University, Xi’an, 710032 China; 2 Department of Otolaryngology-Head and Neck Surgery, the First Affiliated Hospital of Zhengzhou University, Zhengzhou, 450052 China

**Keywords:** Oxeiptosis, Oxidative stress, Hearing loss, Noise

## Abstract

Noise-induced hearing loss is a prevalent form of sensorineural hearing impairment that negatively impacts quality of life and has no effective clinical treatments. Damage due to oxidative stress in cochlear hair cells is thought to be the typical pathological basis. Oxeiptosis is oxidative stress-induced, caspase-independent modality of cell death. In this study, we found that oxeiptosis plays an important role in noise-induced hearing loss, which has not been previously identified. Using protein quantification, protein-protein interaction studies, and immunofluorescence staining in cellular models, we elucidated the pivotal molecules of oxeiptosis. Building on the *in vitro* experimental data, we detected characteristic protein alterations along the oxeiptosis pathway in noise-induced hearing loss murine models. Furthermore, the pharmacological suppression effectively attenuated noise-induced oxeiptosis in cochlear hair cells and partially alleviated hair cell death. This study confirms the existence of a new cell death pathway in NIHL and provides a potential treatment alternative.

**Supplementary Information:**

The online version contains supplementary material available at 10.1007/s12264-025-01585-z.

## Introduction

Noise is a major occupational hazard worldwide [1]. A 2021 World Health Organization report listed noise as one of the top three causes of hearing loss. The most serious health consequence of occupational noise is noise-induced hearing loss (NIHL), which has a profound impact on individuals of all ages [2]. NIHL is one of the most common types of sensorineural hearing loss. It is usually characterised by elevated hearing thresholds, speech perception, auditory processing disorders, and accompanying symptoms, including tinnitus and hyperacusis [3–6]. It is also associated with a range of non-auditory symptoms such as sleep disorders, impairment of cognitive competence, cardiovascular diseases, and other neurasthenic syndromes [2, 7]. The main pathological basis of NIHL is the loss of cochlear hair cells, which cannot regenerate after injury. Excessive noise exposure induces the accumulation of intracellular reactive oxygen species (ROS), calcium dyshomeostasis, reduces mitochondrial membrane potential, and leads to hair cell necrosis and apoptosis [8, 9]. Additionally, research has shown that noise may trigger not only cell necrosis and apoptosis but also other types of regulated cell death (RCD), including ferroptosis. These key modulators or crosstalk molecules in the signal network provide new avenues for the treatment of NIHL [10–12]. However, there are currently no effective clinical treatments for NIHL. It remains unclear whether hair cells die in other modalities.

Oxidative stress-induced damage to hair cells is thought to be the main pathological basis of NIHL [13]. The accumulation of pathological ROS leads to irreversible conformational changes in macromolecules such as proteins, lipids, and DNA, leading to structural and functional abnormalities in organelles, such as the mitochondria and endoplasmic reticulum, and ultimately loss of cell integrity [14–16]. However, little is known about the diverse signalling pathways involved in the oxidative stress that leads to cell death. Oxeiptosis has recently been described as a caspase-independent, apoptosis-like, ROS-induced cell death modality. This mode of cell death involves Kelch-like ECH-associated protein-1 (KEAP1), phosphoglycerate mutase 5 (PGAM5), and the interaction between apoptosis-inducing factor mitochondria-associated 1 (AIFM1) [16]. Oxeiptosis is not mediated by FAS/FASL death receptors, nor does it involve the mitochondrial and endoplasmic reticulum stress-mediated apoptotic pathways. Oxeiptosis does not exhibit specific cellular or histomorphological characteristics but rather mediates a completely different signalling pathway. It relies on KEAP1 to initiate the cell death process, dephosphorylate AIFM1 through PGAM5, and induce cell death [17].

KEAP1 is the main intracellular ROS-monitoring sensor [18]. Under physiological conditions, KEAP1 ubiquitinates and degrades nuclear factor erythroid-derived 2-like 2 (NRF2), a transcription factor that drives the expression of cytoprotective antioxidant genes. An increase in intracellular ROS levels leads to oxidation of KEAP1, which prevents its ability to degrade NRF2. Consequently, NRF2 accumulates and translocates into the nucleus, inducing the expression of antioxidant factors such as NAD(P)H quinone dehydrogenase 1 (NQO1), haemoxygenase-1 (HO-1) , and thioredoxin (TXN) [19]. However, this protective mechanism fails to respond to prolonged or severe oxidative stress and may ultimately result in cellular damage and death [20]. Studies have reported that under high ROS concentrations, KEAP1 may functionally switch from cytoprotection to induction of cell death through its dissociation from PGAM5 [21]. PGAM5 is a mitochondrial outer membrane protein widely distributed in humans. It anchors to the outer mitochondrial membrane via its N-terminal mitochondrial targeting sequence, whereas its C-terminal phosphatase domain remains exposed in the cytoplasm [22]. In the oxeiptosis, after disassociation from the ternary complex of KEAP1, PGAM5 and NRF2, PGAM5 is internalised into the mitochondrial lumen to dephosphorylate its substrate AIFM1 at a highly conserved serine residue at position 116 [17]. AIFM1 is a mitochondrial inner-membrane-anchored protein necessary for maintaining mitochondrial respiratory chain complex I in healthy cells [23]. After receiving the stimuli of cell death signal, AIFM1 is cleaved by calpains and then released from the mitochondria into the cytoplasm and transported to the nucleus by chromatin concentration and massive DNA disruption, resulting in cell death [24, 25]. In oxeiptosis, dephosphorylation at ser116 of AIFM1 leads to cell death; however, the specific mechanism by which dephosphorylated AIFM1 induces oxeiptosis in cells remains unclear. A proteomics study involving 53,026 individuals listed a detailed atlas of 2,920 plasma proteins related to diseases and 986 health-related traits, revealing that plasma AIFM1 is linked to sensorineural hearing loss [26]. Large-scale proteomic studies can refine our understanding of health and disease and enable precision medicine. The role of AIFM1 and related cell death pathways in hearing loss-related diseases requires further investigation.

**Fig. 1:**
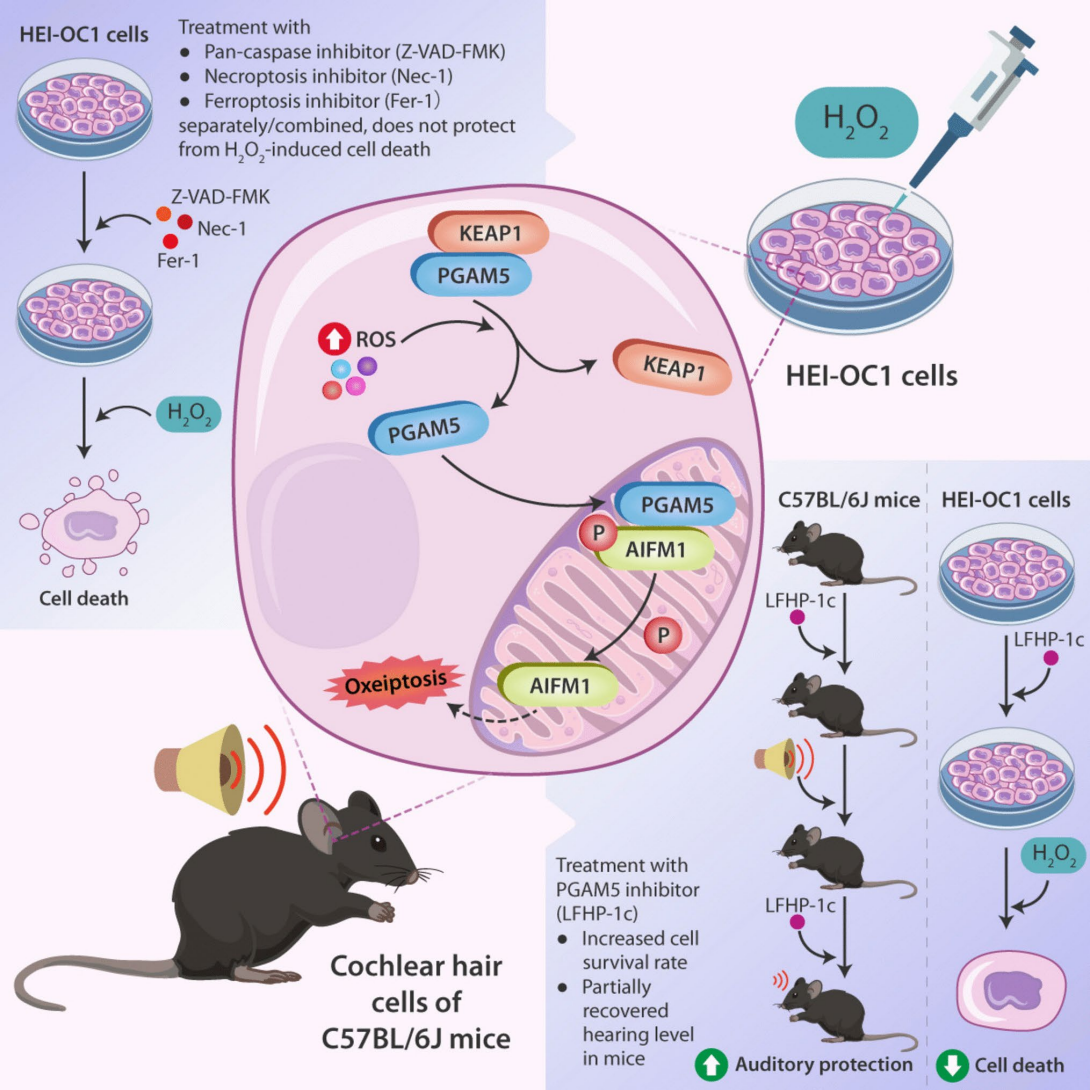
Oxeiptosis plays an important role in NIHL. Using protein quantification, protein-protein interaction studies, and immunofluorescence staining in HEI-OC1 cell models, we elucidated the pivotal molecules of oxeiptosis. Building on the *in vitro* experimental data, we subsequently detected characteristic protein alterations along the oxeiptosis pathway in NIHL murine models. The pharmacological suppression of PGAM5 (LHPF-1c) effectively attenuated noise-induced oxeiptosis in cochlear hair cells and partially alleviated hair cell death. The abbreviations KEAP1, PGAM5, and AIFM1 stand for Kelch-like ECH-associated protein-1, phosphoglycerate mutase 5, and apoptosis-inducing factor mitochondria-associated 1, respectively. Additionally, ROS is an acronym for reactive oxygen species.

In the present study, we found that hydrogen peroxide (H_2_O_2_)-treated mouse cochlear HEI-OC1 cells could not be rescued after treatment with specific inhibitors of necrosis, apoptosis, and ferroptosis, suggesting that there may be other oxidative stress-related death pathways in NIHL. We detected changes in the expression and binding relationship of KEAP1, PGAM5, and AIFM1 in the oxeiptosis signalling pathway *in vitro*, and the intervention of PGAM5 activity suggested its promotion of cochlear hair cell oxeiptosis under oxidative stress. At the same time, alterations in related proteins were also detected in mouse cochlear tissue after noise exposure. Injection of PGAM5 inhibitors before noise exposure in mice partially alleviated hair cell death and hearing threshold decline. Taken together, our results show that oxidative stress can induce hair cells oxeiptosis in NIHL (Fig. 1).

## Materials and Methods

### Cell Culture

The inner ear cell line HEI-OC1 was purchased from UCLA Technology Development Group (Los Angeles, CA, United States). It was cultured in Dulbecco’s modified Eagle’s medium (DMEM;C1199500, Gibco, Grand Island, USA) with 10% fetal bovine serum (10099141, Gibco, Grand Island, USA) and 50 μg/mL ampicillin (Sangon Biotech, Shanghai, China) at 33 °C in a humidified 5% CO_2_ atmosphere. After the 24 h culture reached 70–80% concentration, H_2_O_2_ was added to the medium to induce oxidative stress.

### Animal Studies

All animals were purchased from the Experimental Animal Center of Air Force Military Medical University. All experimental protocols met the requirements and were approved by the Institutional Animal Protection and Utilization Committee of the Air Force Military Medical University (IACUC-202500122). All efforts were made to minimise suffering and to reduce the number of animals used.

Each animal was placed in a round wire cage with a diameter of 9 cm and given food and water as usual. The animals were exposed to a broadband noise of 2 to 20 kHz at a sound pressure level of 105 decibels for 2 h. The sound exposure chamber generates noise by driving a speaker (model 2450H, JBL, California, USA) through a power amplifier (AX-500U, Yamaha, Japan). Electronic audio documents were generated and manipulated using audio editing software. The noise exposure levels at various locations within the sound chamber were calibrated, and the sound consistency during exposure was verified using a sound level meter (AWA6292, Aihua, Hangzhou, China). The control group was not subjected to any noise exposure. The *in vivo* experiment was performed with 8-week-old male C57BL/6J mice, and 18 mice were randomly divided into 3 groups after the auditory brainstem response (ABR) test. The PGAM5 inhibitor was dissolved in a mixture of PEG300, Tween 80, and saline, as instructed. In the PGAM5 inhibitor group, 2 mg/kg of inhibitor was intraperitoneally injected 2 h before noise and again injected 2 h after noise. The noise group was injected with the same dose of solvent, and the control group was injected with the same dose of solvent without noise or other treatment. The ABR test was performed 1 and 14 days later. The WB and tissue staining samples from the control group and the experimental group were collected on the 1st day and 14th day, respectively, for subsequent experiments.

### Plasmid Transfection

HEI-OC1 cells were inoculated into six-well plates at a density of 2 × 10^5^ cells per well and cultured until reaching 80–90% confluency. 1h before transfection, the medium was replaced with Opti-MEM reduced serum medium (31985070, Thermo Fisher, Waltham, USA). For transfection complex preparation, 2.5 μg plasmid DNA was mixed with 5 μL P3000™ Enhancer (L3000015, Thermo Fisher) in 125 μL Opti-MEM serum-free medium and gently pipetted. In a separate tube, 5 μL Lipofectamine 3000 reagent (L3000015, Thermo Fisher) was combined with 125 μL Opti-MEM and incubated at room temperature for 5 min. The DNA-P3000 mixture was then combined with the Lipofectamine 3000 solution at a 1:1 volume ratio, vortexed gently, and incubated for 15 min at room temperature. The 250 μL transfection complexes were added dropwise to the cell culture medium, followed by gentle swirling of the plate to ensure an even distribution. After 6 h of incubation, the medium was replaced with serum-containing complete medium. Transfection efficiency was assessed 24–48 h post-transfection using fluorescence microscopy for EGFP expression. The schematic diagram of the pcDNA3.1-3xFlag vector is shown in the Supplementary Material (Additional File 2). Untransfected cells and empty vector controls (pcDNA3.1) were included as negative controls. Antibiotic-free medium was used throughout to avoid cytotoxicity. Transfection efficiency was detected by quantitative real-time PCR. All primer sequences are presented in the Supplementary Material (Table S1).

### Cell Viability Assay

Cell viability was detected using enhanced cell counting kit-8 (CCK-8) (IC-1519, Incellgene, Xi’an, China). After treatment, 100 μL CCK-8 working solution was added into each well of the 96-plate and incubated for 1 h at 37°C, before the absorbance of each well at 450 nm was read using a microplate reader.

### Lactate Dehydrogenase (LDH) Assay

HEI-OC1 cells were inoculated into 96-well plates and treated according to their respective experimental group for the corresponding time. The cell supernatant was then taken and incubated according to the manufacturer’s instructions. The absorbance of each hole was then read at 490 nm to detect the activity of LDH in the cell culture medium.

### Calcein AM/PI Staining

HEI-OC1 cells were inoculated into Confocal Petri dishes (801002, NEST, USA) at the appropriate density and cultured until reaching experimental requirements. The culture medium was removed, and the cells were gently washed twice with pre-warmed phosphate buffer solution (PBS) to eliminate dead cell debris. The staining working solution was prepared by adding 5 μL of PI solution (KGA9501, KeyGEN Biotech, Nanjing. China) into 10 mL of Opti-MEM reduced serum medium, followed by vortex mixing in the dark. Then, 5 μL of Calcein AM solution was added to the diluted PI solution. Thereafter, 1 mL of staining solution was added to each dish and incubated while protected from light at 37°C for 30 min. After staining, the cells were visualised using an Olympus FV3000 confocal microscope system: viable cells exhibited a green fluorescence (Calcein AM ex/em: 490/515 nm, KGA9501, KeyGEN Biotech, Nanjing, China) while dead cells showed a red fluorescence (PI ex/em: 535/617 nm ( KGA9501, KeyGEN Biotech, Nanjing, China). All procedures were performed under light-protected conditions, and detection was completed within 2 h post-staining to ensure fluorescence stability.

### ATP Concentration

ATP is a critical indicator of cellular metabolism and a hallmark molecule of metabolically active cells. ATP content detection is used to measure cell viability by quantifying ATP levels. HEI-OC1 cells were inoculated into 96-well plates and cultured until reaching experimental requirements. After treatment according to experimental groups and incubation at 37°C for 24 h, the CellTiter-Lumi™ Luminescent Cell Viability Assay solution (C0065, Beyotime, Shanghai, China) was added to each well and incubated for 10 min. Chemiluminescence was measured using a multimode microplate reader equipped with chemiluminescence detection capabilities. The relative cell viability was calculated directly based on the chemiluminescence readings.

### ROS Measurement

Confocal Petri dishes were inoculated with HEI-OC1 cells. Once the cells were attached to the dishes, they were cultured for 20 min in the dark in 5 μmol/L DCFH-DA (GC30006, GLPBIO, Montclair, USA) to detect total ROS and 500 nmol/L Mito-SOX Red (M36007, Thermo Fisher) to detect mitochondrial ROS. The cells were incubated with Hoechst reagent (C1028, Beyotime) for 10 min, and images were acquired using a fluorescence microscope and processed using ImageJ software.

### Western Blot and Co-Immunoprecipitation (Co-IP)

Protein was extracted from cells by using RIPA buffer (R0278, Sigma-Aldrich, Germany) containing 1% phosphatase inhibitors, protease inhibitors, and phenylmethylsulfonyl fluoride after transfecting cells with a Flag-tagged KEAP1 overexpression plasmid. Pre-cleared lysates were then incubated with prepared anti-Flag Magnetic Beads (M8823, Sigma-Aldrich) for 4 h at 4°C with end-over-end rotation. After incubation, the solution was separated on a magnetic frame for 10 s, the supernatant was removed, and the beads were washed three times and heated at 95 °C for 5 min for hot elution. Proteins pulled down by Anti-Flag Magnetic Beads were used to detect KEAP1-binding proteins. For endogenous protein co-immunoprecipitation, anti-AIFM1 antibody (1:50) or normal mouse IgG (1:50) control was added to the TBS to make the antibody working solution. The protein A+G magnetic beads (P2180, Beyotime) were added to the antibody working solution or the normal mouse IgG working solution. Then the beads were turned over and incubated on a flipping mixer at room temperature for 1 h. Binding Protein A + G beads of antibody or normal IgG were added to pre-cleared lysates and incubated at 4 °C overnight for co-immunoprecipitation. After incubation, the beads were washed three times and heated at 95 °C for 5 min for hot elution. Protein concentration was measured by the BCA Protein Assay Kit (13222, Cowin Bio, Taizhou, China). For the western blot, HEI-OC1 cells were lysed using RIPA lysis buffer containing 1% phosphatase inhibitors, protease inhibitors, and phenylmethylsulfonyl fluoride. The samples were then centrifuged at 12,000×g and 4°C for 30 min. The supernatant was drained into new EP tubes and quantified using a BCA Protein Assay Kit (13222, Cowin bio) and heated at 95 °C for 5 min.

Each sample was then loaded into the respective lanes of the gel, separated by 10% sodium dodecyl sulfate-polyacrylamide gel electrophoresis and transferred onto a polyvinylidene fluoride membrane (IPVH00010, Millipore, Germany). The blots were incubated for 1 h in blocking buffer containing 5% bovine serum albumin in 0.1% Tween 20 in phosphate buffer solution (PBS-T) and then incubated overnight at 4 °C with the corresponding primary antibody diluted in blocking buffer. The primary antibody included KEAP1 rabbit antibody (8047, Cell Signalling Technology, Massachusetts, USA), PGAM5 rabbit antibody (HPA036978, Thermo Fisher Scientific), phospho-AIFM1 (Ser-116) (AP5501, ECM Biosciences, Kentucky, USA), AIFM1 rabbit antibody (17984-1-AP, Proteintech, Wuhan, China), and β-actin mouse antibody (66009-1-Ig, Proteintech). The blots were washed in PBS-T, incubated for 2 h at 25 °C with a peroxidase-conjugated secondary antibody, and developed using an enhanced chemiluminescence reagent (34577, Thermo Fisher Scientific, Waltham, USA). Immunoreactive bands were visualized using a chemiluminescence system (Touch Imager, e-BLOT, Shanghai, China).

### Immunofluorescent Staining

For the cellular immunofluorescence assay, cells underwent sequential processing as follows: Initial fixation was performed with 4% paraformaldehyde, followed by membrane permeabilization using 0.5% Triton X-100. After three PBS washes, nonspecific binding sites were blocked with blocking buffer containing 5% bovine serum albumin for 1 h at 37 °C. Primary antibody incubation was carried out overnight at 4 °C using KEAP1 (8047, Cell Signalling Technology, Massachusetts, USA) and PGAM5 rabbit (HPA036978, Thermo Fisher Scientific) monoclonal antibodies. Following another PBS washing cycle, cells were exposed to species-matched fluorescent secondary antibodies (SA00001-2, Proteintech) for 1 h at ambient temperature. To enable multiplex detection, a second blocking step preceded overnight incubation with COX IV mouse monoclonal antibodies (11967, Cell Signalling Technology), which were subsequently detected with corresponding secondary reagents. Nuclear counterstaining was achieved through 5-min DAPI treatment (HY-D1738, MedChemExpress, New Jersey, USA) under light-protected conditions. All procedural stages incorporated three PBS washes between each step. Fluorescent signals were ultimately visualized using an Olympus FV3000 confocal microscope system.

For the cochleae immunofluorescence assay, C57BL/6J mice were euthanized by cervical dislocation for cochlear isolation. The dissected cochlear specimens underwent sequential processing: The cochleae were fixed with 4% paraformaldehyde for 24 h and decalcified with 10% sodium EDTA solution for 48 h. The dissected cochleae were blocked with 5% bovine serum albumin at room temperature for 1 h after being rinsed with 0.1% PBS-T three times. Then, they were incubated with KEAP1 (8047, Cell Signalling Technology) and PGAM5 rabbit (HPA036978, Thermo Fisher Scientific). The samples were washed with PBS-T for 5 min three times before being incubated with secondary antibodies at room temperature for 1 h. The secondary antibodies included the Alexa Fluor 647-conjugated donkey anti-rabbit or mouse IgG (A-31573, A-31571, Invitrogen, California, USA) and the Alexa Fluor 488-conjugated donkey anti-rabbit or mouse IgG (A-21206, R37114, Invitrogen). After the samples were incubated with the secondary antibodies and washed again with PBS-T, they were mounted with an antifade mounting medium. Fluorescent signals were also visualized using an Olympus FV3000 confocal microscope system.

### ABR Measurement

ABR thresholds were used to evaluate the hearing function of mice. Briefly, the mice were anaesthetized by intraperitoneal injection of a mixture of ketamine (100 mg/kg) and xylazine (10 mg/kg) (Sangon Biotech, Shanghai, China)and placed in a sound-isolated chamber. The RZ6 system (Tucker-Davies Technologies) was used to detect mouse ABR thresholds. Mice were put on a 37°C thermostatic heating pad after being anaesthetized. Electrodes were inserted into the vertex and subdermally behind the ears to record the ABRs. A broadband speaker placed in front of the mouse ear was used to produce the sound stimulus. Five frequencies (4, 8, 16, 24, and 32 kHz) were used for the ABR test. At each frequency, the test measured from a 90 dB sound pressure level, which was defined as the ABR threshold in this experiment, and then progressively decreased by 5 dB until wave I disappeared to reduce error.

### Statistical Analyses

Data presented in the text and figures are the means and standard errors of the mean (mean ± SD). One-way analysis of variance and Dunnett’s multiple comparison test were used to compare the data among groups, whereas a paired sample *t* test was used to analyse differences within the same group. Statistical analyses were performed using GraphPad Prism 10 and SPSS 23.0. *P* values less than 0.05 (*P* < 0.05) indicate statistical significance.

## Results

### Caspase-Independent Cell Death Pathway Exists in HEI-OC1 Cells Under Oxidative Stress in Addition to Apoptosis, Necroptosis, and Ferroptosis

**Fig. 2:**
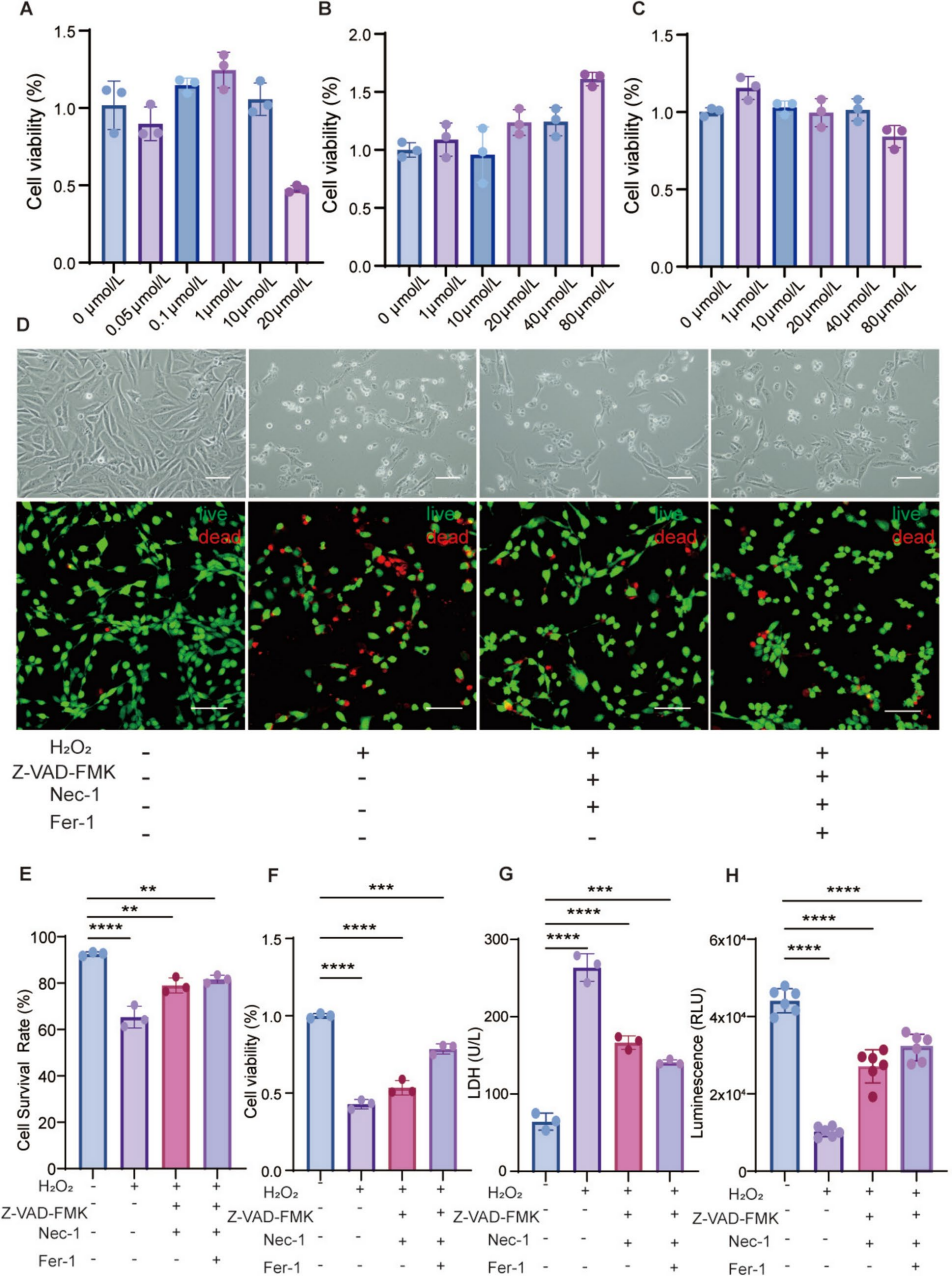
Caspase-independent cell death pathway exists in HEI-OC1 cells under oxidative stress in addition to apoptosis, necroptosis and ferroptosis. **A** CCK-8 assay detected the safety concentration of Z-VAD-FMK that does not affect the cell viability of HEI-OC1 cells. **B** CCK-8 assay detected the safety concentration of Nec-1 that does not affect the cell viability of HEI-OC1 cells. **C** The safety concentration of Fer-1 was detected using the CCK-8 assay. **D** After treatment with two or three inhibitors and H_2_O_2_, the cell morphology was observed, and the survival rates were detected by live/dead cell staining assay. Scale bar, 100 μm. Z-VAD-FMK is a kind of pan-caspase inhibitor. Nec-1 and Fer-1 represent necroptosis inhibitor and ferroptosis inhibitor respectively. **E** Comparison and statistical analysis of cell survival rates after treatment with two or three inhibitors, *n =* 6. **F** Cell activity was detected by CCK-8, *n =* 6. **G** The released LDH activity in HEI-OC1 cells was determined by the LDH release assay, *n =* 6.** H** Detection of ATP content in cells after different treatments. All the data are presented as the mean ± SD of three independent experiments. One-way ANOVA was used to compare the treatment group with the control group, *n =* 6.**, *P* < 0.01, ***, *P* < 0.001, ****, *P* < 0.0001 compared with the control.

To investigate the ROS-mediated cell death pathways in cochlear hair cells associated with NIHL, we treated HEI-OC1 cells with H_2_O_2_. HEI-OC1 cells exposed to H_2_O_2_ exhibit morphological alterations and reduced cell viability. First, we treated HEI-OC1 cells with varying concentrations of the pan-caspase inhibitor Z-VAD-FMK or necroptosis inhibitor Nec-1 to determine the concentrations of Z-VAD-FMK and Nec-1 that did not affect baseline cell viability (Fig. 2A, B). Based on previously published concentrations and our viability data, we selected 10 μmol/L Z-VAD and 20 μmol/L Nec-1 for 2-h pre-treatment in subsequent experiments. Studies have also demonstrated that ferroptosis contributes to NIHL. In alignment with the reported literature and our concentration screening, we chose 40 μmol/L ferroptosis inhibitor Fer-1 for the subsequent experiments (Fig. 2C). The results showed that pre-treatment with Z-VAD-FMK and Nec-1 failed to completely abrogate H_2_O_2_-induced cytotoxicity. Similarly, the combined application of a pan-caspase inhibitor (Z-VAD-FMK), necroptosis inhibitor (Nec-1), and ferroptosis inhibitor (Fer-1) failed to fully rescue H_2_O_2_-induced HEI-OC1 cell death. Compared to the control group, the triple inhibitor combination failed to fully restore cellular morphology (Fig. 2D).

We further assessed cell survival using a live/dead cell staining assay, in which dead cells were labelled with red fluorescence (Propidium Iodide) and viable cells were labelled with green fluorescence (Calcein-AM). The results demonstrated a significant increase in the number of dead cells following H_2_O_2_ treatment compared to the controls. Although cell death was reduced in both the Z-VAD-FMK + Nec-1 pre-treatment group and the triple inhibitor (Z-VAD-FMK + Nec-1 + Fer-1) pre-treatment group, neither intervention restored cell viability to baseline control levels (Fig. 2D, E). Similar results were observed in the CCK8 (Fig. 2F). The LDH content increased significantly after H_2_O_2_ treatment, but in the Z-VAD-FMK + Nec-1 pre-treatment group and the triple inhibitor pre-treatment group, neither intervention restored the LDH content to the baseline control level (Fig. 2G). We also determined the ATP content, which was proportional to cell activity. These results were consistent with previous findings (Fig. 2H).

By assessing cell membrane integrity, cellular metabolic activity, and energy metabolism, we found that H_2_O_2_-induced HEI-OC1 cell damage could not be rescued by cell death inhibitors. Neither pre-treatment with Z-VAD-FMK, Nec-1, nor a combination of Z-VAD-FMK, Nec-1, or Fer-1 fully restored cell viability following exposure. Collectively, our data suggest that H_2_O_2_-induced HEI-OC1 cell death is mediated through a pathway independent of caspases, ferroptosis, or necroptosis.

### Oxidative Stress Induces KEAP1-PGAM5 Dissociation in HEI-OC1 Cells Initiating Oxeiptosis

**Fig. 3:**
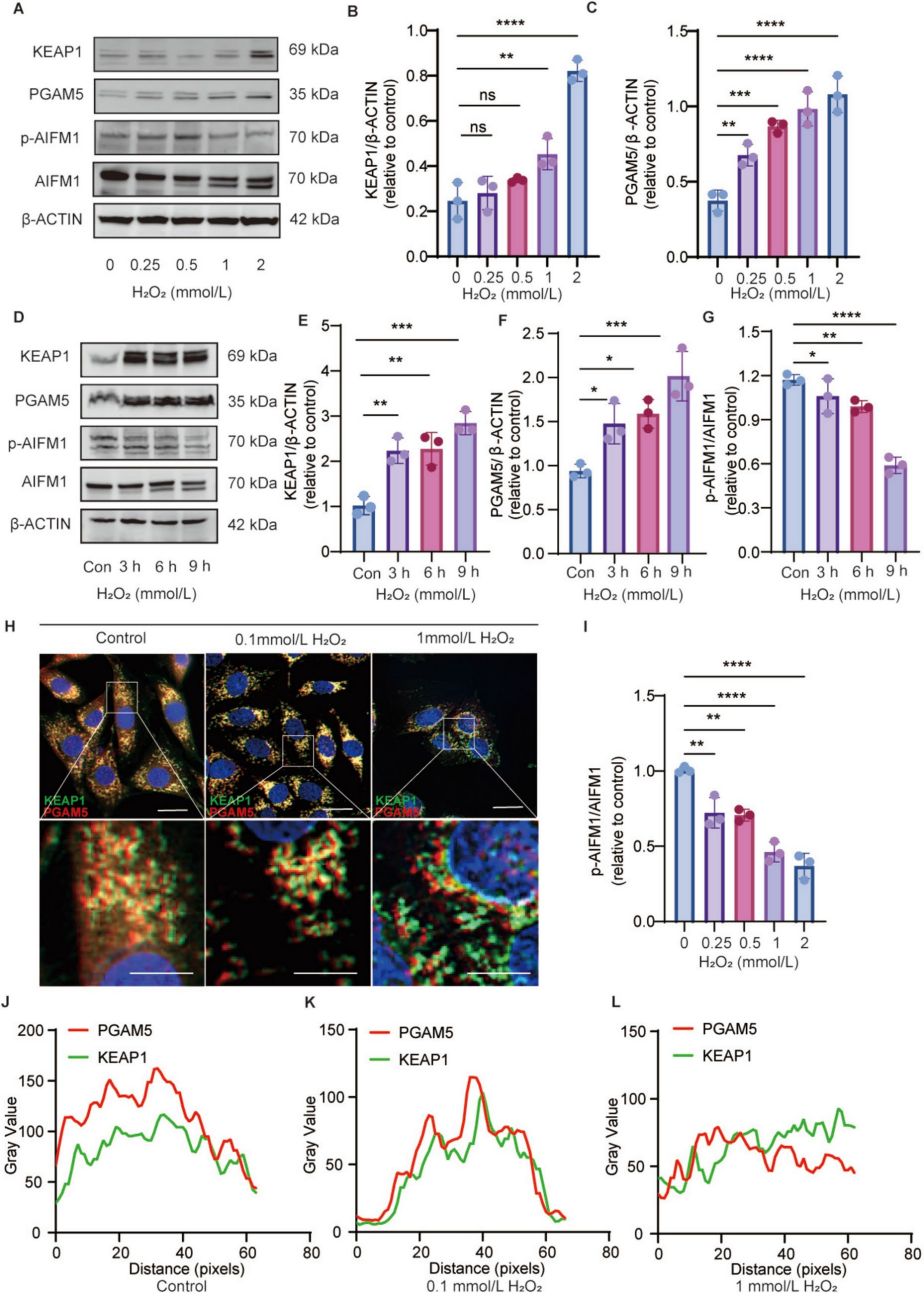
Oxidative stress induces changes in the expression of the protein related to the oxeiptosis pathway and triggers KEAP1-PGAM5 dissociation in HEI-OC1 cells, initiating oxeiptosis. The abbreviations KEAP1, PGAM5, and AIFM1 stand for Kelch-like ECH-associated protein-1, phosphoglycerate mutase 5, and apoptosis-inducing factor mitochondria-associated 1, respectively. CON represents the control group. **A** KEAP1, PGAM5, AIFM1, and p-AIFM1 protein expression at different concentrations was detected by western blot, using β-actin as the endogenous housekeeping control gene. **B** Comparison and statistical analysis of western blot results of KEAP1 protein, fold change relative to 0 mmol/L (control), *n =* 3. **C** Comparison and statistical analysis of Western blot results of PGAM5 protein, fold change relative to 0 mmol/L (control), *n =* 3. **D** KEAP1, PGAM5, AIFM1, and p-AIFM1 protein expression at different timepoints (0.5 mmol/L) was detected by western blot, using β-actin as the endogenous housekeeping control gene. **E, F, G** Comparison and statistical analysis of western blot results of KEAP1, PGAM5, AIFM1, and p-AIFM1protein, fold change relative to control, *n =* 3. **H** Localisation relationship was detected by immunofluorescence staining. Scale bar, 25 μm. Scale bar in the magnified image is 10 μm. **I** Comparison and statistical analysis of western blot results of p-AIFM1 protein, fold change relative to 0 mmol/L (control), *n =* 3. **J, K, L** The qualitative analyses of co-localisation of KEAP1 and PGAM5. All the data are presented as the mean ± SD of three independent experiments. One-way ANOVA was used to compare the treatment group with the control group. ns means non-significant, *P < 0.05, **P < 0.01, ***P < 0.001, ****P < 0.0001 compared with the control.

Oxeiptosis is a recently proposed caspase-independent modality of cell death that is triggered by oxidative stress. Considering that oxidative stress is a major pathogenic factor of NIHL and based on our previous results, we aimed to explore whether oxidative stress causes oxeiptosis in hair cells in a cellular model. According to previous studies, oxeiptosis signalling pathways include KEAP1-PGAM5-AIFM1, and dephosphorylation of AIFM1 Ser116 is a marker and basic molecule of the oxeiptosis signalling pathway [17]. After treating HEI-OC1 cells with different concentrations of H_2_O_2_, we observed that when the H_2_O_2_ concentration was greater than 0.5 mmol/L, the protein related to the oxeiptosis pathway changed significantly. We found that the expression of KEAP1 and PGAM5 was upregulated after H_2_O_2_ treatment (above 0.5 mmol/L) (Fig. 3A–C), whereas the expression of phosphorylated AIFM1 decreased (Fig. 3I). This change preliminarily verified that oxidative stress might lead to oxeiptosis in cochlear hair cells. Next, 0.5 mmol/L H_2_O_2_ was used to treat the cells, and changes in protein levels were detected at different time points. The oxeiptosis level increased with treatment time (Fig. 3D–G). In addition, the dissociation of PGAM5 from KEAP1 is a prerequisite for oxeiptosis. After the cells were treated with H_2_O_2_, changes in localisation were detected by immunofluorescence staining. Our experimental data demonstrated that PGAM5 and KEAP1, which exhibit constitutive co-localisation under physiological conditions, undergo stimulus-dependent spatial dissociation following specific activation. This phenomenon was not observed in the 0.1 mmol/L treatment group (Fig. 3H). The qualitative analyses of co-localisation are shown(Fig. 3J–L). Based on these findings, we propose that oxeiptosis is activated in hair cells under high levels of oxidative stress.

**Fig. 4:**
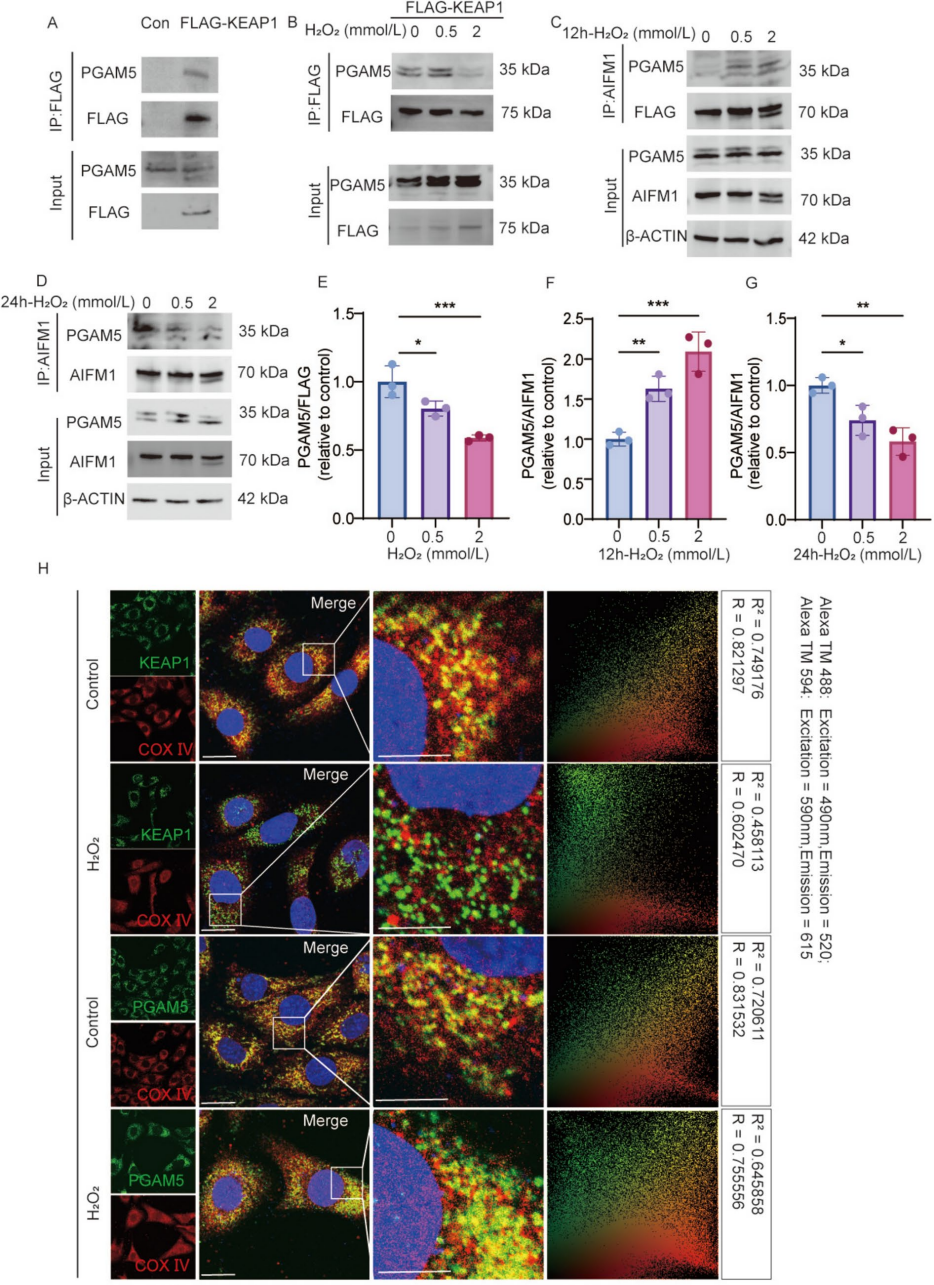
Oxidative stress induces changes in the co-location relationship of the KEAP1-PGAM5 and PGAM5-AIFM1. **A** Co-IP was used to detect the interaction between KEAP1 and PGAM5. **B** Co-IP results showed that PGAM5 bound to KEAP1 was reduced in H_2_O_2_-stimulated HEI-OC1 cells compared to that in the control group. **C** Co-IP results showed that the combination of PGAM5 and AIFM1 increased after 12 h of H_2_O_2_ treatment. **D** Co-IP results showed that the binding of PGAM5 and AIFM1 decreased after 24 h of H_2_O_2_ treatment. **E** Comparison and statistical analysis of western blot results in B, fold change relative to control, *n =* 3. **F, G** Comparison and statistical analysis of western blot results in **C, D**, fold change relative to control, *n =* 3. The statistical differences between the treatment group and the control group were examined using one-way ANOVA. *P < 0.05, **P < 0.01, ***P < 0.001 compared with the control. **H** Immunofluorescence staining revealed the co-localisation relationship of KEAP1 and PGAM5 with mitochondria. Scale bar, 25 μm. Scale bar in the magnified image is 10 μm. The abbreviations KEAP1 and PGAM5 stand for Kelch-like ECH-associated protein-1 and phosphoglycerate mutase 5. COX IV (Cytochrome c Oxidase IV) is a mitochondrial marker protein. Statistical analysis was performed using Pearson correlation analysis, where *R* represents the correlation coefficient, and *R*^2^ (*R*-squared) denotes the coefficient of determination.

Subsequently, we performed Co-IP experiments to confirm the interaction between KEAP1 and PGAM5. HEI-OC1 cells were transiently transfected with an overexpression plasmid encoding Flag-tagged KEAP1 (Fig. S1A, B). Following 48-h transfection and lysis, Co-IP experiments showed that there is a binding relationship between the two proteins (Fig. 4A). Then, PGAM5 protein levels associated with KEAP1 were detected, and their binding relationships were analyzed. The results showed that PGAM5 bound to KEAP1 was reduced in H_2_O_2_-stimulated HEI-OC1 cells compared to that in the control group (Fig. 4B, E). In addition, we found that KEAP1 and PGAM5 exhibit constitutive mitochondrial co-localisation under physiological conditions. Mitochondria were labelled with COX IV (red fluorescence), whereas KEAP1 and PGAM5 were individually tagged with green fluorescence. Notably, KEAP1 translocated to the cytoplasm (*R*_control_ = 0.82, *R*_noise_ = 0.60), whereas PGAM5 retained its mitochondrial residence upon oxidative stress stimulation (*R*_control_ = 0.83, *R*_noise_ = 0.75) (Fig. 4H). Based on these findings, we conclude that high concentrations of H_2_O_2_ induce the dissociation of PGAM5 from KEAP1 in HEI-OC1 cells, thereby initiating the first step of oxeiptosis.

### Oxidative Stress Induced the Dissociation of PGAM5 From AIFM1 and Promoted the Dephosphorylation of AIFM1

By treating HEI-OC1 cells with varying concentrations of H_2_O_2_, we found that phosphorylated AIFM1 expression decreased when H_2_O_2_ concentrations exceeded 0.5 mmol/L. Consequently, we selected 0.5 mmol/L for the subsequent experiments using HEI-OC1 cells. We performed a magnetic bead pull-down assay targeting the AIFM1 protein and quantified its bound PGAM5 using a Co-IP experiment. Reduced PGAM5 binding to AIFM1 was observed, leading to AIFM1 dephosphorylation (Fig. 4C). AIFM1 dephosphorylation is a hallmark of oxeiptosis. Studies have reported that PGAM5 possesses phosphatase activity, which enables it to remove phosphate groups from substrate molecules and modulate downstream biological effects. In H_2_O_2_-treated HEI-OC1 cells, oxidative stress triggers KEAP1-PGAM5 dissociation, liberating PGAM5 to execute its constitutive phosphatase function and directly mediating AIFM1 dephosphorylation.

When analysing the combined relationship between PGAM5 and AIFM1, we observed transient interaction potentiation at 12 h post-stimulation, followed by progressive complex dissociation at 24 h (Fig. 4C, D, F, and G). We hypothesised that this dynamic change was related to the phosphatase activity of PGAM5. Initial substrate engagement during dephosphorylation requires enhanced enzyme-substrate interactions to facilitate phosphomonoester bond hydrolysis, followed by obligatory complex disassembly upon phosphate group liberation. The phosphatase activity of PGAM5 further promotes the dephosphorylation of AIFM1, thus completing the key step of oxeiptosis in HEI-OC1 cells.

In summary, our findings elucidate the mechanism underlying AIFM1 dephosphorylation during oxeiptosis and demonstrate that oxidative stress induces PGAM5-AIFM1 dissociation in HEI-OC1 cells, thereby promoting AIFM1 dephosphorylation and triggering oxeiptosis.

### Inhibiting PGAM5 Phosphatase Activity Reduces HEI-OC1 Cells Oxeiptosis

**Fig. 5:**
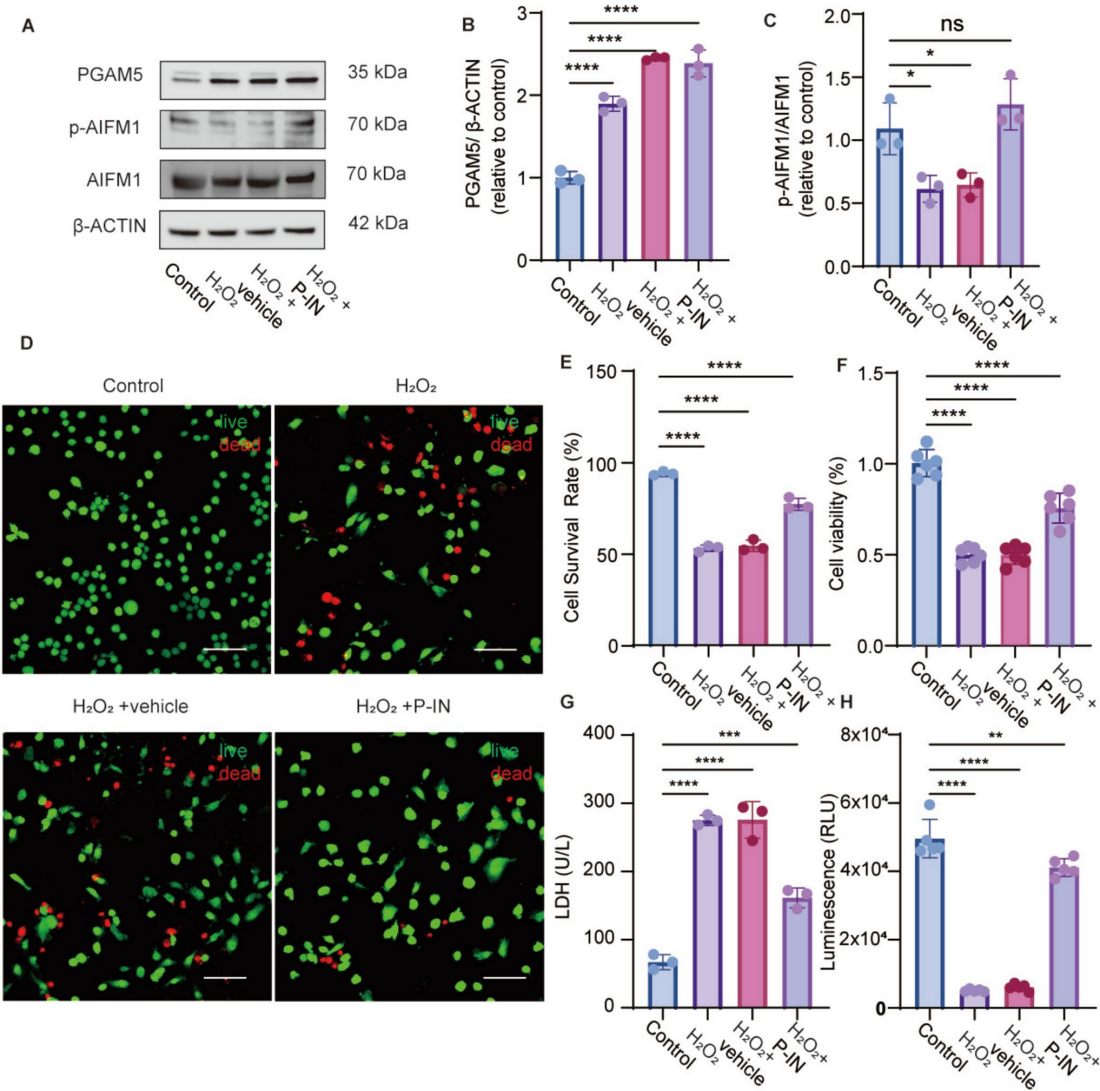
Inhibiting PGAM5 phosphatase activity reduces HEI-OC1 cells oxeiptosis. PGAM5 represents phosphoglycerate mutase 5 and AIFM1 represents apoptosis-inducing factor mitochondria-associated 1. P-IN is the abbreviation of PGAM5 inhibitor. **A** Western blot result of protein related to oxeiptosis after PGAM5 inhibitor treatment. **B, C** Comparison and statistical analysis of western blot results of PGAM5 and p-AIFM1 protein, fold change relative to control, *n =* 3. **D** The survival rate after PGAM5 inhibitor treatment was detected by live/dead cell staining assay. Scale bar, 100 μm. **E** Comparison and statistical analysis of cell survival rates after treatment with PGAM5 inhibitor, *n =* 3. **F** Cell activity was detected by CCK-8, *n =* 6. **G** The released LDH activity was determined by the LDH release assay, *n =* 3. **H** Detection of ATP content in cells after PGAM5 inhibitor treatments. All the data are presented as the mean ± SD of three independent experiments, *n =* 5. One-way ANOVA was used to compare the treatment group with the control group. ns means non-significant, *P < 0.05, **P < 0.01, ***P < 0.001, ****P < 0.0001 compared with the control.

The above results demonstrate that the dephosphorylation of AIFM1 is led by the activity of PGAM5 phosphatase, which is a crucial step in oxeiptosis. Next, we examined the effect of inhibiting PGAM5 phosphatase activity on oxeiptosis modulation in HEI-OC1 cells. LFHP-1c is a selective PGAM5 inhibitor (P-IN) that competitively binds to its catalytic domain to block its enzymatic functions. It has been reported to protect blood-brain barrier integrity against ischaemic injury and exhibit neuroprotective activity in ischaemic stroke. Prior to H_2_O_2_ treatment, we pretreated HEI-OC1 cells with LFHP-1c to attenuate the phosphatase activity of PGAM5. After 24 h of continued treatment, western blot analysis revealed that AIFM1 phosphorylation levels were elevated in the inhibitor group compared to the controls (Fig. 5A–C). Additionally, live/dead cell staining was performed to assess cell survival. Results showed that compared with the control group, the P-IN group exhibited higher cell survival rates and a more intact and healthier cellular morphology following H_2_O_2_ exposure (Fig. 5D, E). Subsequently, we evaluated cell viability using the CCK-8 assay and found that the P-IN group exhibited significantly improved cell viability after H_2_O_2_ treatment (Fig. 5F). We also assessed LDH release and intracellular ATP levels, revealing that LFHP-1c pre-treatment mitigated H_2_O_2_-induced cytotoxicity, as evidenced by enhanced cellular viability in the P-IN group compared to the H_2_O_2_-only controls (Fig. 5G, H).

**Fig. 6:**
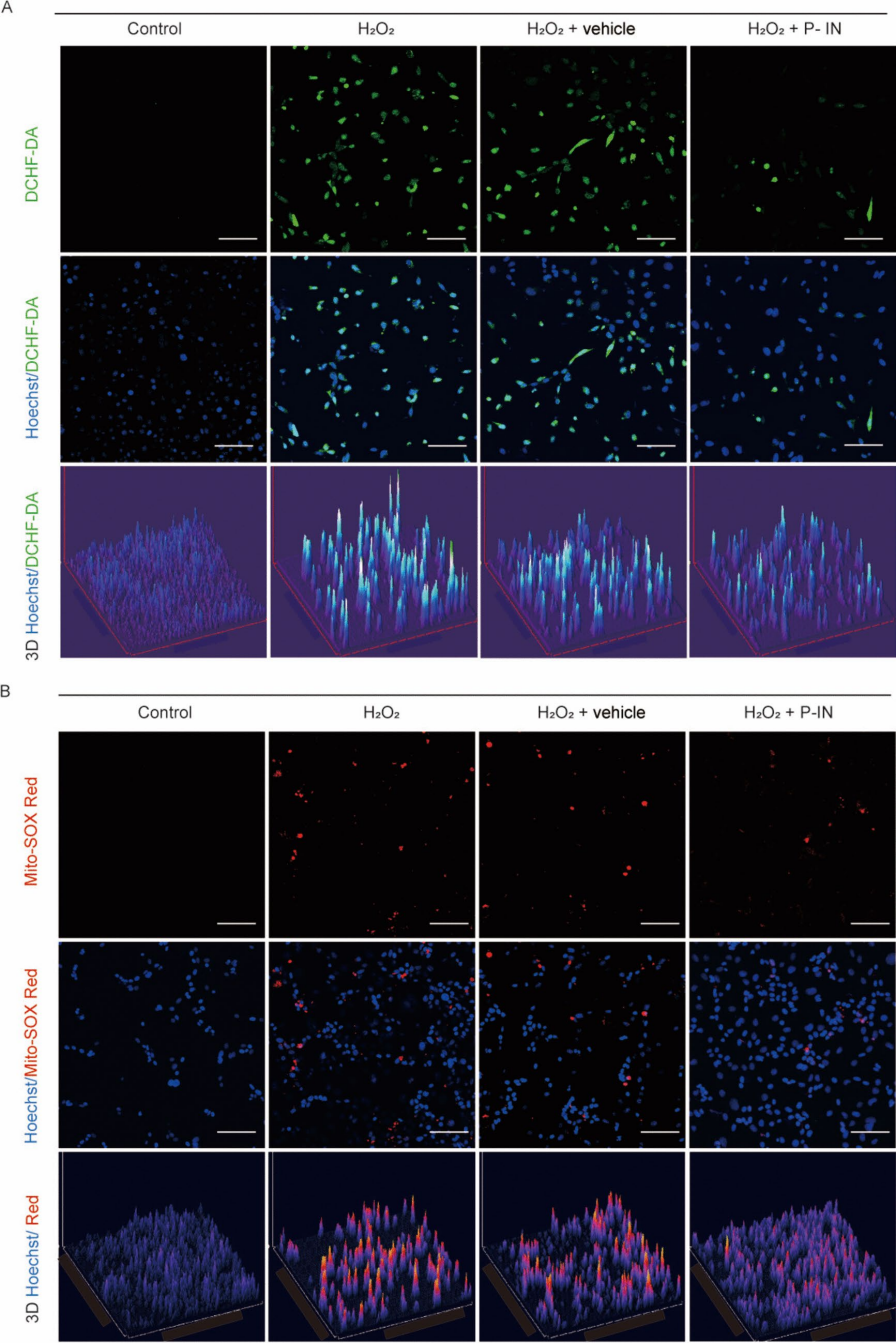
The levels of ROS in cells and mitochondria decreased after treatment with PGAM5 inhibitors. P-IN is the abbreviation of PGAM5 inhibitor. **A** DCFH-DA staining showed that PGAM5 inhibitor treatment significantly reduced intracellular ROS accumulation. **B** Mito-SOX Red staining showed that PGAM5 inhibitor treatment also reduced ROS accumulation. Scale bar, 100 μm.

We demonstrated that after treatment with the inhibitors, the level of oxeiptosis decreased and the cell survival rate increased. The antioxidant activity was assessed to elucidate the potential mechanism underlying its protective effects in HEI-OC1 cells. We used DCFH-DA to probe total ROS in the cells and Mito-SOX to probe mitochondrial ROS. Treatment with H_2_O_2_ elevated both intracellular and mitochondrial ROS levels, whereas cellular and mitochondrial ROS were significantly weakened in the P-IN inhibitor group (Fig. 6A, B). These results suggest that LFHP-1c reduced apoptosis in HEI-OC1 cells and protected them from oxidative stress by maintaining mitochondrial function and limiting the burst production of ROS.

### Noise Exposure Induced Oxeiptosis in Mice Cochlear Hair Cells

**Fig. 7:**
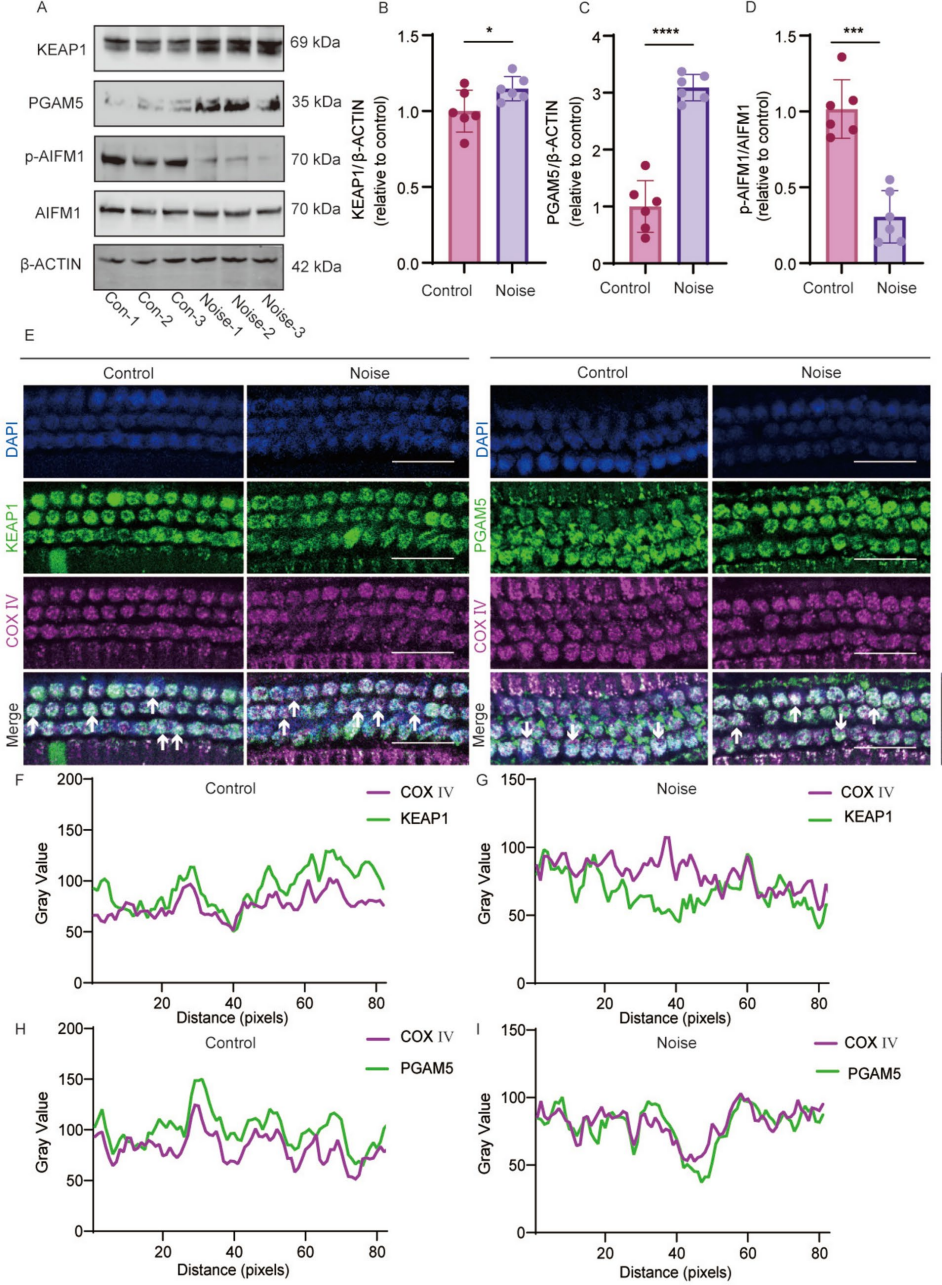
Noise exposure induced oxeiptosis in mice cochlear hair cells. The abbreviations KEAP1 and PGAM5 stand for Kelch-like ECH-associated protein-1 and phosphoglycerate mutase 5. COX IV (Cytochrome c Oxidase IV) is a mitochondrial marker protein. CON represents the control group. **A** The western blot results showed the changes of oxeiptosis-related proteins in the cochlea of mice after noise. **B–D** Comparison and statistical analysis of western blot results of PGAM5 and p-AIFM1 protein, fold change relative to control, *n =* 6. All the data are presented as the mean ± SD of three independent experiments. The statistical differences between the treatment group and the control group were examined using *t* test. *P < 0.05, ***P < 0.001,****P < 0.0001 compared with the control. **E** Immunofluorescence analysis of the basilar membrane revealed coordinated localisation of KEAP1 and PGAM5. Scale bar, 25 μm. Arrows indicate missing hair cells. **F** The qualitative analyses of co-localisation of KEAP1 and COX IV in the control group. **G** The qualitative analyses of co-localisation of KEAP1 and COX IV in the noise group. **H** The qualitative analysis of co-localisation of PGAM5 and COX IV in the control group. **I** The qualitative analysis of co-localisation of PGAM5 and COX IV in the noise group. Arrows indicate the representative hair cells.

Our previous data demonstrated that oxidative stress induces oxeiptosis in HEI-OC1 cells, and the inhibition of PGAM5 phosphatase activity reduces oxeiptosis while enhancing cell viability. Subsequent *in vivo* validation showed elevated KEAP1 and PGAM5 expression in noise-exposed mouse cochleae compared to controls, accompanied by decreased levels of phosphorylated AIFM1 (Fig. 7A–D). Immunofluorescence analysis of the basilar membrane revealed coordinated localisation of KEAP1 and PGAM5. Co-localisation analysis using green (KEAP1/PGAM5) and purple (COX IV) markers demonstrated reduced mitochondrial co-localisation of KEAP1 after noise exposure, whereas PGAM5 localisation remained unchanged, indirectly confirming the initial phase of oxeiptosis (Fig. 7E–I).

**Fig. 8:**
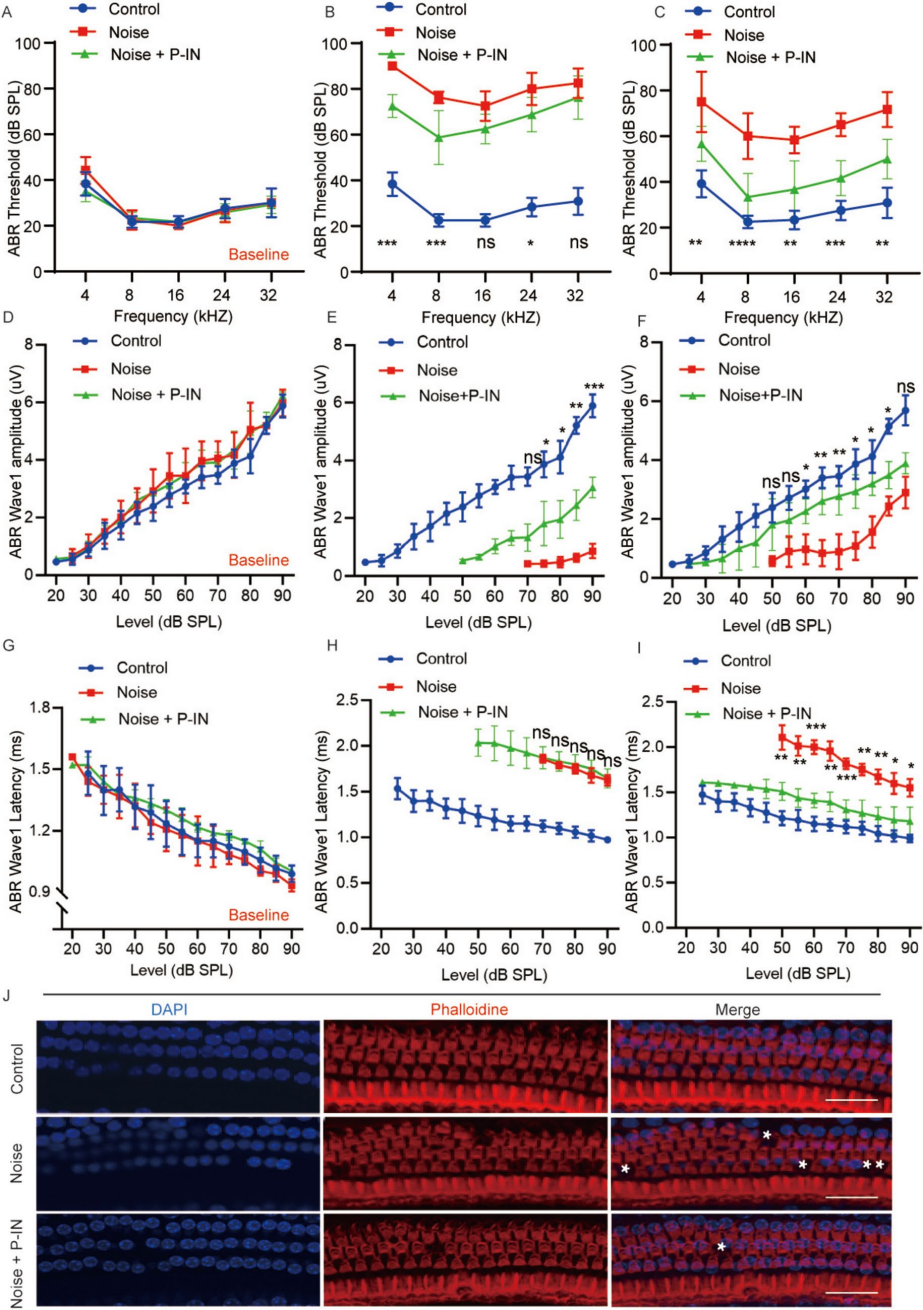
Inhibiting PGAM5 phosphatase activity mitigates oxeiptosis, thereby preserving the auditory function. P-IN is the abbreviation of PGAM5 inhibitor. **A** Baseline levels of ABR threshold in mice, *n =* 5. **B, C** ABR threshold results in mice on days 1 and 14 after noise exposure, *n =* 5. **D** Baseline levels of ABR Wave I amplitude in mice, *n =* 5. **E, F** ABR Wave I amplitude results in mice on day 1 and 14 after noise exposure, *n =* 5. **G** Baseline levels of ABR Wave I latency in mice. **H, I** ABR Wave I latency results in mice on days 1 and 14 after noise exposure, *n =* 5. All the data are presented as the mean ± SD of three independent experiments. Two-way ANOVA was used to assess significance. *P < 0.05, **P < 0.01, ***P < 0.001, ****P < 0.0001 (Noise vs. Noise + P-IN). **J** Immunofluorescence staining of hair cells from mice on Day 14 after noise exposure. Hair cells were stained with phalloidin (red), scale bar, 25 μm. * represents missing hair cells.

To investigate the role of AIFM1 dephosphorylation in cochlear hair cell oxeiptosis, C57BL/6J mice were treated with an LFHP-1c inhibitor (P-IN) or vehicle before and after noise exposure. Auditory function can be measured by determining the auditory brainstem response (ABR). ABR threshold at 1 and 14 days post-exposure revealed comparable hearing thresholds between the groups on day 1, but significantly better auditory protection at every frequency in the P-IN group at later time points (Fig. 8A–C). ABR wave I amplitudes and latency are depicted between the two groups to further evaluate auditory function. A significant recovery of ABR wave I amplitude and latency was found between the two groups on day 1 (Fig. 8E, H) and day 14 (Fig. 8F, I). Immunofluorescence analysis confirmed that the inhibitor treatment attenuated outer hair cell loss in noise-damaged mice (Fig. 8J). Quantification of OHCs survival at the middle turn in was shown(Fig. S1F). These findings indicate that noise-induced cochlear oxeiptosis can be mitigated by inhibiting AIFM1 dephosphorylation, thereby preserving the auditory function.

In our current study, we generated *Aifm1*-p.S115A mutation mice. Preliminary results indicate a decline in auditory function in these mice (Fig. S1C–E). We hypothesise that the S115A mutation abolishes phosphorylation at the S115 site of AIFM1, leading to spontaneous oxeiptosis in cochlear cells and subsequent auditory impairment. However, the phenotypic characteristics of hearing loss require further exploration, and additional auditory data need to be collected. These findings demonstrate the involvement of oxeiptosis in NIHL, yet its functional role and mechanistic pathways require further elucidation.

## Discussion

A growing number of novel RCD pathways have been identified and are increasingly associated with various human physiological pathologies. Necroptosis and pyroptosis are observed during development and during viral infections [27]. Ferroptosis, entotic cell death, netotic cell death, parthanatosis, and lysosome-dependent cell death are associated with cellular stress responses and self-protective mechanisms. Multiple RCD modalities of RCD have been identified in auditory studies. Previous studies have reported that NIHL is associated with apoptosis, necroptosis, and ferroptosis, suggesting that noise exposure activates cell death via multiple pathways. The oxidative stress caused by high-intensity noise can simultaneously trigger mitochondria-dependent apoptosis [28], necroptosis, and ferroptosis [29], ultimately leading to hair cell damage. Aminoglycoside antibiotic-induced ototoxicity is primarily associated with apoptosis and necroptosis. Its ototoxic mechanisms involve mitochondrial dysfunction and ROS accumulation, which activate apoptosis through the c-Jun N-terminal kinase (JNK) pathway and necroptosis via mixed lineage kinase domain-like protein (MLKL) phosphorylation, thereby inducing cochlear hair cell death [30]. Cisplatin exposure activates receptor-interacting protein kinase-1 (RIPK1) and RIPK3, which are key regulators of necroptosis, leading to membrane permeabilisation and cell lysis [31]. In addition, cisplatin can cause ferroptosis in hair cells by increasing the expression of NCOA4 and microtubule-associated protein 1 light chain 3- II (LC3-II) and inhibiting glutathione peroxidase (GPX) [29]. Hair cells are not the sole target. Pharmacological agents also induce multiple forms of RCD in the stria vascularis (SV) and its margins. Activation of the NOD-like receptor thermal protein domain-associated protein 3 (NLRP3) inflammasome mediates cisplatin-induced pyroptosis in cochlear SV and marginal cells. During ageing, mitochondrial dysfunction and exacerbated oxidative stress may preferentially activate p53-dependent apoptotic pathways as well as dysregulate iron metabolism and lipid peroxidation [32], resulting in the progressive loss of hair cells. In this study, we confirmed the existence of a new modality for RCD in HEI-OC1 cells under oxidative stress. HEI-OC1 cells exhibit activation of the KEAP1-PGAM5-AIFM1 signalling pathway under oxidative stress, which is a hallmark of RCD called oxeiptosis.

Previous studies have demonstrated that necroptosis and ferroptosis are involved in the pathogenesis of NIHL; however, the exact pathogenic mechanisms remain unclear. There are currently no effective clinical treatments for NIHL. In 2018, Holze *et al.* [17] proposed oxeiptosis as a caspase-independent ROS-induced cell death pathway. In our *in vitro* studies, we validated that oxidative stress triggers oxeiptosis in HEI-OC1 cells, which is characterised by activation of the KEAP1-PGAM5-AIFM1 signalling axis. Moreover, pharmacological disruption of these molecules increases cell survival rates. These findings suggest that inhibiting oxeiptosis enhances cellular resistance to oxidative stress and further reduces cell death, highlighting its therapeutic potential for NIHL. We observed the same phenomenon in the mouse models. Noise-exposed mice exhibit altered expression of oxeiptosis-associated molecules in the cochlea. Compared with the noise-only treatment group, mice that received noise exposure combined with PGAM5 inhibitor administration demonstrated better hearing thresholds. Our findings are consistent with those of recent studies, indicating that oxidative stress triggers oxeiptosis in other tissues and cells. Oxeiptosis has been reported to contribute to the pathogenesis of several diseases. In intervertebral disc degeneration, mitochondrial damage in nucleus pulposus cells elevates intracellular ROS levels, and inhibition of oxeiptosis slows the progression of lumbar disc degeneration [33]. In vitiligo research, melanocytes from patients exhibit hallmark features of oxeiptosis [34, 35]. Conversely, in hepatocellular carcinoma, activation of oxeiptosis suppresses tumour cell growth [36]. Additionally, oxeiptosis may play a role in diabetic endothelial cell dysfunction and the development of diabetic cardiomyopathy [37]. Our study revealed that noise exposure induces oxeiptosis in hair cells, identifying this as a potential mechanism underlying noise-induced cochlear damage.

Oxidative stress refers to the imbalance between the production and clearance of ROS within an organism or cell, leading to ROS accumulation and subsequent oxidative damage. Under physiological conditions, approximately 1% of oxygen is converted to ROS through processes such as mitochondrial oxidative phosphorylation and enzymatic redox reactions [38]. However, excessive ROS levels can induce cellular damage, including protein oxidation, lipid peroxidation, and DNA strand breaks, ultimately triggering cell death. High-intensity noise exposure generates substantial ROS in the cochlea [13]. Excessive ROS disrupt the mitochondrial membranes and respiratory chain proteins in hair cells, resulting in mitochondrial DNA (mtDNA) mutations, lipid peroxidation, and protein oxidation, which collectively contribute to cochlear dysfunction [38]. Mitochondrial dysfunction exacerbates cellular energy deficits and amplifies ROS production, ultimately leading to irreversible hearing loss [39, 40]. Excessive ROS levels also induce apoptosis in cochlear hair cells. Beyond triggering apoptosis via the activation of caspase family proteases, studies have shown that ROS activates AMPK in outer hair cells (OHCs), a key cellular energy sensor activated by metabolic stress, which mediates OHC death through ROS/AMPK-dependent pathways [8]. ROS play pivotal roles as critical signalling molecules in the initiation, progression, and resolution of inflammatory responses. Research has demonstrated that ROS induces early upregulation of pro-inflammatory cytokines, facilitating rapid infiltration of inflammatory cells and exacerbating post-noise hair cell injury. In addition to directly inducing inflammation, ROS modulate the expression of inflammation-related genes, further regulating inflammatory cascades within hair cells [41–43]. Furthermore, the cochlea contains resident immune cells such as macrophages, which, upon activation, express various inflammatory mediators and participate in the early stages of cochlear inflammatory responses. In the present study, oxeiptosis was activated only in HEI-OC1 cells exposed to high ROS concentrations. Similarly, animal experiments revealed no detectable changes in oxeiptosis-associated proteins following low-intensity noise exposure. Therefore, we propose that oxeiptosis is a new mechanism of hair cell death specific to high-intensity noise injury. Despite extensive research on ROS-induced hair cell damage and death, the underlying mechanisms remain complex and poorly understood.

As a newly identified cell death modality triggered by oxidative stress, oxeiptosis has been reported not only for its pathogenic potential but also for its critical role in maintaining redox homeostasis, acting as a ROS-triggered “safe elimination mechanism.” In pulmonary epithelial cells, oxeiptosis clears damaged cells while avoiding inflammatory cascades, potentially serving as an adaptive mechanism against environmental toxins [17]. Early studies have suggested that oxeiptosis may exert tumour-suppressive effects by eliminating precancerous cells, although its regulation within the tumour microenvironment and interplay with immune responses remain to be elucidated. Furthermore, while it is well established that ROS include highly oxidative species, such as the superoxide anion (O_2_^‒^), hydroxyl radical (OH^‒^), singlet oxygen (_1_O^2^), hydrogen peroxide (H_2_O_2_), and ozone (O_3_), whether specific ROS subtypes selectively activate oxeiptosis requires further investigation.

NRF2 is a critical factor in the antioxidant response. As a key modulator of NRF2, KEAP1 promotes NRF2 ubiquitination and proteasomal degradation. Studies have shown that the KEAP1/NRF2 axis can modulate cellular susceptibility to ferroptosis [44, 45]. However, in this study, KEAP1 switched its role to PGAM5 dissociation, thereby activating oxeiptosis in hair cells. Consequently, it remains unclear whether oxeiptosis and ferroptosis share an interactive regulatory network. Furthermore, PGAM5 promotes apoptosis under severe oxidative stress. In models of intrinsic apoptosis induced by arenobufagin and staurosporine, PGAM5 recruits Drp1 and Bax to the mitochondrial outer membrane, forming a ternary complex that triggers mitochondrial fission, induces mitochondrial outer membrane permeabilisation, and ultimately activates apoptosis [46]. Studies have suggested that the role of PGAM5 in determining cell fate is context-dependent and is influenced by stress intensity and its interaction with molecular chaperones [47]. In our study, PGAM5 exerted phosphatase activity to dephosphorylate AIFM1. AIFM1 encodes an apoptosis-inducing factor (AIF), which was initially identified as a mediator of caspase-independent apoptosis [48]. Subsequent studies showed that AIF is critical for parthanatos, an RCD modality that shares features of apoptosis and necrosis [49]. Besides, *AIFM1* gene mutations cause auditory neuropathy in humans. Tao Shi *et al*. generated an *Aifm1* p.R450Q knock-in mouse model (KI) based on the human *Aifm1* p.R451Q mutation [50]. Hemizygote KI male mice exhibited progressive hearing loss and a gradual reduction in the number of spiral ganglion neuron cells [50]. Our findings indicate that AIFM1 participates in a new RCD mechanism underlying noise-induced cochlear hair cell loss. Thus, the function of AIFM1 in oxeiptosis, as well as its other biological roles in hair cells and spiral ganglion neuron cells, requires further exploration.

The discovery of oxeiptosis complements the understanding of the modality of ROS-responsive regulated death; however, its molecular details and pathological associations need to be systematically resolved. Among the noise-induced regulated death modes of hair cells, oxeiptosis, as a new mode of cell death, provides a broader prospect for further exploration and elucidation of the mechanism of hair cell injury and death. However, the role of oxeiptosis in oxidative stress-related hearing loss remains to be explored. It remains unclear whether there are still unknown patterns of hair cell death in noise-induced hearing loss. Future studies should integrate single-cell sequencing, chemical biology tools, and disease organoid models to reveal the specific regulation of oxeiptosis and lay the foundation for the development of precision treatment strategies based on oxeiptosis regulation.

Although we found a new cell death pathway in NIHL and provided a potential alternative for the treatment of NIHL, its specific molecular modulation mechanism is not clear. Our *in vitro* findings indicate that oxeiptosis occurs only when oxidative stress reaches a specific threshold. Future studies should investigate whether varying degrees of noise are associated with different levels of oxeiptosis. Additionally, given the current lack of clinical samples, further experimental validation is required to substantiate our findings.

## Supplementary Information

Below is the link to the electronic supplementary material.

Supplementary file1 (PDF 696 KB)
