# Supplementary material for: Unveiling a Novel Mechanism in Noise-Induced Hearing Loss: Oxeiptosis-Mediated Regulated Cell Death of Cochlear Hair Cell
Source: Neurosci Bull. 2026 Feb 16;42(7):1473–91. doi: 10.1007/s12264-025-01585-z (PMC13388591; doi:10.1007/s12264-025-01585-z)
Supplement: Supplementary file 1 — Supplementary file1 (PDF 696 KB) [file 12264_2025_1585_MOESM1_ESM.pdf]

Supplementary Materials

Additional file 1

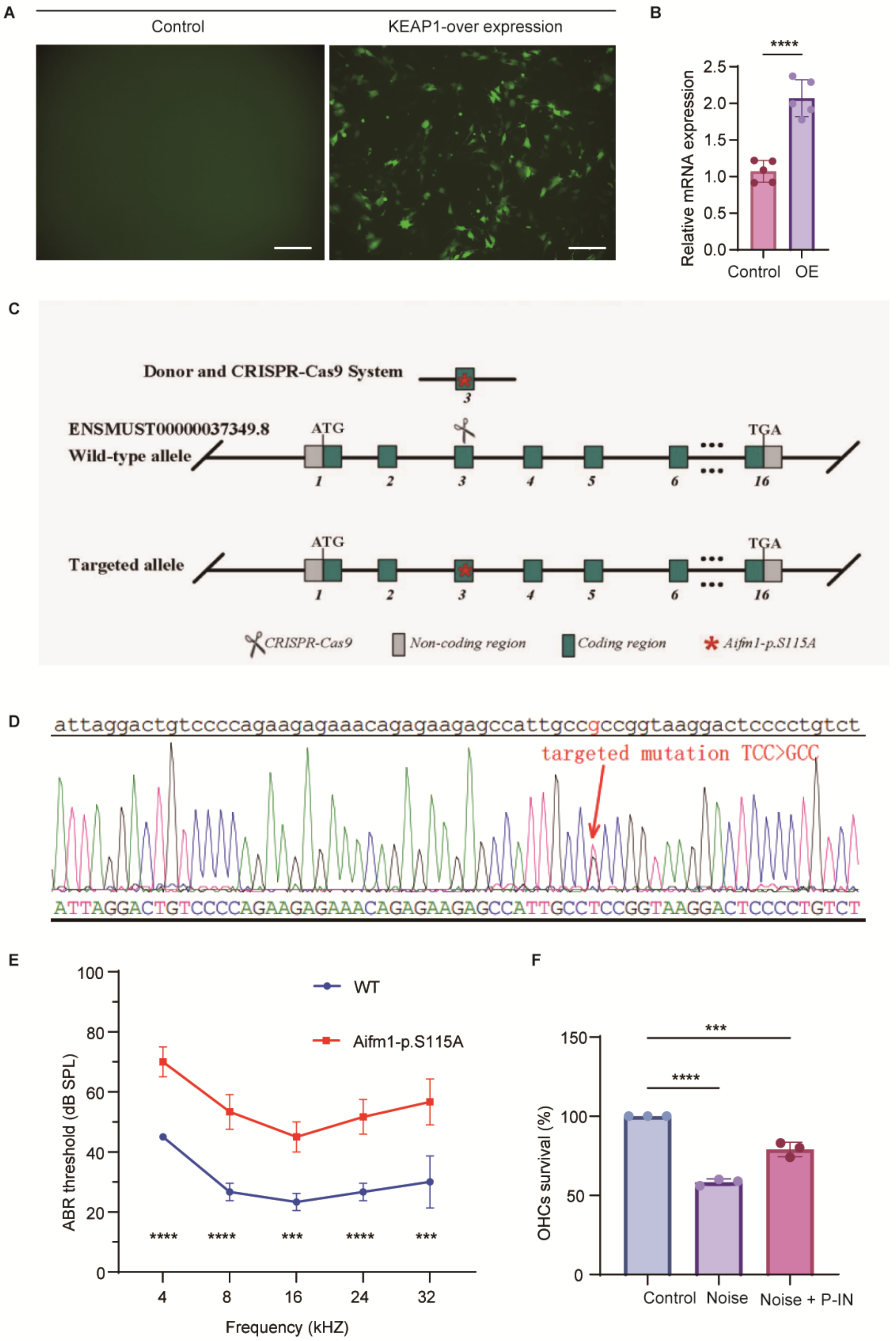

Fig. S1 Construction of *Aifm1*-p.S116A mutant mice. **A** GFP fluorescence was monitored to assess transfection efficiency following Keap1-overexpression plasmid delivery. Scale bar, 200 μm. **B** Quantitative real-time PCR analysis of Keap1 expression after overexpression (OE), $n = 5$. **C** CRISPR-Cas9-mediated gene-editing strategy for generating *Aifm1*-p.S115A mutant mice. **D** Sequencing validation of *Aifm1*-p.S115A mutant mice. **E** Baseline levels of auditory brainstem response (ABR) threshold in *Aifm1*-p.S115 mutant mice. Multiple *t*-tests were performed to assess significance. **F** Quantification of OHCs survival at the middle turn in Fig. 7J. P-IN is the abbreviation of PGAM5 inhibitor. One-way ANOVA was used to compare the treatment group with the control group. All the data are presented as the mean ± SD of three independent experiments ($n = 3$). *$P < 0.05$, **$P < 0.01$, ***$P < 0.001$,****$P < 0.0001$ compared with the control.

Additional file 2 — The primers used for qRT-PCR experiments

Table 1. qRT-PCR primers list

Names	Sequences
Keap1 (mouse)	Forward: 5′- TGCCCCTGTGGTCAAAGTG-3′
	Reverse: 5′- GGTTCGGTTACCGTCCTGC-3′
Gapdh (mouse)	Forward: 5′- GTCTTCACCACCATGGAGAA -3′
	Reverse: 5′- TAAGCAGTTGGTGGTGCAG-3′

Additional file 3
The plasmid used for cell transfection
The schematic diagram of the pcDNA3.1-3xFlag vector is shown below.

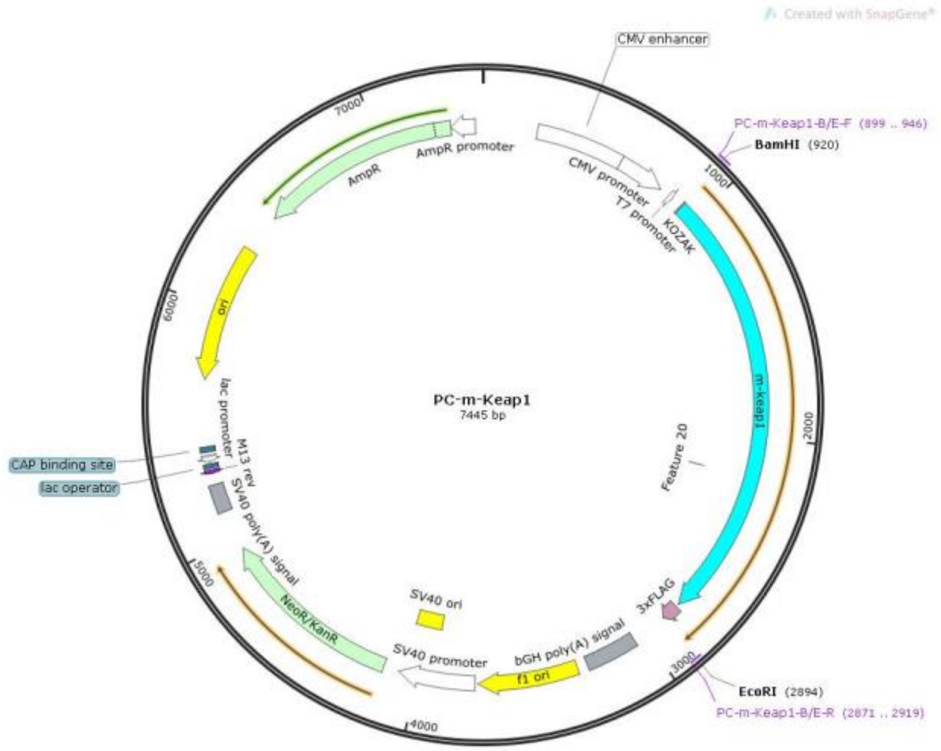

Plasmid sequencing results：

AGACCCACTGCTTACTGGCTTATCGAAATTAATACGACTCACTATAGGGAGACCCAAGC
TGGCTAGTTAAGTTGGTACCGAGCTCGGATCCGCCACCATGCAGCCCGAACCCAAGCTT
AGCGGGGCTCCCCGCAGCAGCCAGTTCCTGCCCCTGTGGTCAAAGTGCCCCGAGGGGGC
CGGGGACGCAGTGATGTATGCCTCCACGGAGTGCAAGGCAGAGGTGACGCCCTCGCAG
GACGGTAACCGAACCTTCAGCTACACACTAGAGGATCACACCAAGCAGGCTTTTGGCGT
CATGAACGAGCTTCGCCTGAGCCAGCAACTCTGTGACGTGACCCTGCAGGTCAAATATG
AGGACATCCCAGCTGCCCAATTCATGGCTCACAAAGTGGTGCTGGCCTCCTCCAGCCCA
GTCTTTAAAGCCATGTTCACCAACGGGCTTCGGGAGCAGGGCATGGAGGTGGTGTCCAT
CGAAGGCATCCACCCTAAGGTCATGGAAAGGCTTATTGAGTTCGCCTACACGGCCTCCA
TCTCCGTGGGCGAGAAGTGTGTCCTGCACGTGATGAACGGGGCGGTCATGTACCAGATT
GACAGCG
TGGTTCGAGCCTGCAGCGACTTCCTCGTGCAGCAGCTGGACCCCAGCAACGCCATTGGC
ATCGCCAACTTCGCGGAGCAGATCGGCTGCACTGAACTGCACCAGCGTGCCCGGGAGTA
TATCTACATGCACTTCGGGGAGGTGGCCAAGCAGGAGGAGTTCTTCAACCTGTCACACT
GCCAGCTGGCCACGCTCATCAGCCGGGATGATCTGAACGTACGCTGCGAGTCCGAGGTG
TTCCACGCGTGCATCGACTGGGTCAAATACGACTGCCCGCAGCGGCGCTTCTACGTGCA
GGCACTGCTGCGGGCCGTGCGCTGCCATGCGCTCACGCCGCGCTTCCTGCAGACGCAGC
TGCAGAAGTGTGAGATCCTGCAGGCCGACGCGCGCTGCAAGGACTACCTGGTGCAGAT

ATTCCAGGAGCTCACGCTGCACAAGCCCACGCAGGCAGTGCCCTGCCGCGCGCCCAAA
GTGGGCCGCCTCATCTACACAGCGGGCGGTTACTTCCGACAGTCGCTCAGCTACCTGGA
GGCCTACAACCCGAGCAATGGCTCCTGGCTGCGCCTGGCCGATCTACAGGTGCCGCGCA
GTGGGCTGGCAGGCTGCGTGGTGGGTGGGCTGCTATACGCTGTGGGCGGCCGCAACAA
CTCTCCGGATGGCAACACTGACTCCAGCGCCCTGGACTGCTACAACCCCATGACCAACC
AGTGGTCGCCCTGTGCCTCTATGAGCGTGCCACGCAACCGCATAGGGGTGGGGGTCATA
GATGGCCACATCTACGCAGTCGGGGGTTCCCACGGCTGCATCCACCACAGCAGCGTGGA
GAGATATGAGCCAGAGCGGGACGAGTGGCATCTAGTCGCGCCAATGTTGACACGGAGG
ATTGGCGTGGGCGTGGCAGTGCTCAACCGCTTGCTGTATGCAGTGGGGGGCTTTGACGG
GACTAACC
GGCTTAACTCCGCAGAATGTTACTATCCAGAGAGGAATGAGTGGCGGATGATCACACCG
ATGAATACCATCCGGAGCGGGGCCGGGGTCTGCGTGCTGCACAACTGTATCTATGCAGC
AGGGGGCTACGATGGGCAGGACCAGTTGAACAGTGTGGAGCGCTACGACGTGGAGACA
GAGACCTGGACTTTCGTAGCCCCCATGAGGCATCACCGTAGTGCGCTGGGGATTACTGT
GCACCAGGGCAAGATCTACGTCCTCGGAGGCTATGATGGCCACACTTTTCTGGACAGTG
TGGAATGCTATGACCCGGACAGTGATACCTGGAGTGAGGTGACCCGCATGACATCTGGC
CGCAGCGGGGTGGGTGTGGCCGTCACCATGGAACCCTGTCGGAAGCAAATTGATCAAC
AAAACTGTACCTGCGGTACCTTAATTAACGACTACAAGGATGACGATGACAAGGATTAC
AAAGACGACGATGATAAGGACTATAAGGATGATGACGACAAATAAGAATTCTGCAGAT
ATCCAGCACAGTGGCGGCCGCTCGAGCATGCATCTAGAGGGCCCTTCGAACAAAAACTC
ATCTCAGAA GAGGATCTGAATATGCATACCGGTCATCATCACCATC
